# Virus/Host Cell Crosstalk in Hypoxic HPV-Positive Cancer Cells

**DOI:** 10.3390/v9070174

**Published:** 2017-07-05

**Authors:** Karin Hoppe-Seyler, Julia Mändl, Svenja Adrian, Bianca J. Kuhn, Felix Hoppe-Seyler

**Affiliations:** 1 Molecular Therapy of Virus-Associated Cancers (F065), German Cancer Research Center (DKFZ), D-69120 Heidelberg, Germany; k.hoppe-seyler@dkfz.de (K.H.-S.); j.maendl@dkfz.de (J.M.); s.adrian@dkfz.de (S.A.); bianca.kuhn@dkfz.de (B.J.K.); 2 Viral Transformation Mechanisms (F030), German Cancer Research Center (DKFZ), D-69120 Heidelberg, Germany

**Keywords:** human papillomavirus, hypoxia, cervical cancer, head and neck cancer, senescence, metabolism, mTOR, therapy

## Abstract

Oncogenic types of human papillomaviruses (HPVs) are major human carcinogens. The expression of the viral *E6*/*E7* oncogenes plays a key role for HPV-linked oncogenesis. It recently has been found that low oxygen concentrations (“hypoxia”), as present in sub-regions of HPV-positive cancers, strongly affect the interplay between the HPV oncogenes and their transformed host cell. As a result, a state of dormancy is induced in hypoxic HPV-positive cancer cells, which is characterized by a shutdown of viral oncogene expression and a proliferative arrest that can be reversed by reoxygenation. In this review, these findings are put into the context of the current concepts of both HPV-linked carcinogenesis and of the effects of hypoxia on tumor biology. Moreover, we discuss the consequences for the phenotype of HPV-positive cancer cells as well as for their clinical behavior and response towards established and prospective therapeutic strategies.

## 1. Introduction

Approximately 50–60% of solid tumors exhibit pronounced hypoxic regions (commonly defined as oxygen concentrations below 1.5–2%) [1,2,3]. Hypoxia is of clinical importance because tumors with higher proportions of hypoxic cells usually have a poor prognosis, including human papillomavirus (HPV)-linked cancers, such as cervical cancers or head and neck squamous cell carcinomas (HNSCCs) [1,4,5]. In the process of HPV-linked carcinogenesis, the viral *E6* and *E7* oncogenes play a central role both for the induction of malignant cell transformation and for the maintenance of the oncogenic phenotype of HPV-positive cancer cells. Recent data indicate that hypoxia has profound effects on the crosstalk between the HPV oncogenes and their host cell, with implications for the malignant phenotype of HPV-positive cancer cells and for their clinical behavior.

## 2. Hypoxia and Cancer

**Figure 1:**
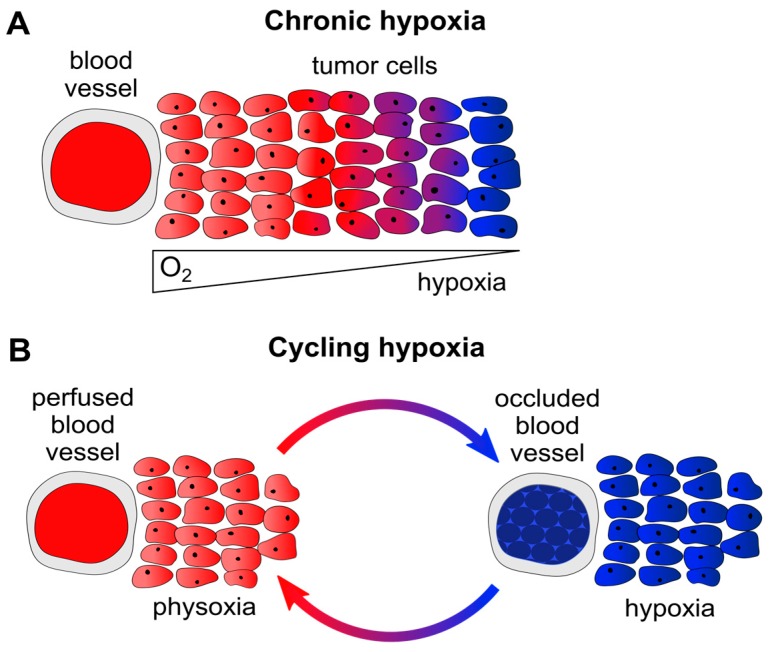
Chronic and cycling hypoxia. (**A**) Diffusion-limited chronic hypoxia due to enlarged distances between tumor blood vessels and tumor cells. Remote tumor cells (>70 µm away from the blood vessel [1]) are inadequately supplied with O_2_ and become hypoxic. Red: oyxgenated tumor cells, blue: hypoxic tumor cells; (**B**) Perfusion-limited cycling hypoxia. Tumor vessels are often abnormally structured and can be temporarily occluded, e.g., through blood cell aggregates. Surrounding tumor cells will be exposed to fluctuating cycles of physoxia (left) or hypoxia (right). Red: oyxgenated tumor cells, blue: hypoxic tumor cells.

In principle, two major forms of hypoxia can be differentiated in solid cancers. Firstly, chronic hypoxia occurs in a time frame of hours to days or weeks. This form of hypoxia is primarily caused by diffusion limitations, e.g., due to enlarged distances (>70 µm [1]) between tumor blood vessels and remote tumor cells (Figure 1A). Secondly, cycling (alternatively called acute, intermittent, or fluctuating) hypoxia primarily results from perfusion limitations, e.g., following the temporary partial or total occlusion of tumor microvessels (which are often structurally and functionally abnormal) through blood cell aggregates (Figure 1B). This latter form of hypoxia exposes tumor cells to repeated cycles of hypoxia and reoxygenation, which can be highly variable in their duration and frequency, for example occurring several times within one hour [1,6,7].

The poor clinical prognosis of hypoxic tumors could, on the one hand, mechanistically be due to hypoxia-linked processes that support the clonal evolution of more malignant tumor cells. These processes include (i) the induction of genetic instability through the downregulation of DNA repair mechanisms [8] and increased production of reactive oxygen species (ROS) [9]; (ii) the metabolic reprogramming of cancer cells, e.g., leading to a higher rate of glycolysis and enhanced lactate production and excretion [10,11]; (iii) the inhibition of cellular tumor suppressor pathways, such as apoptosis [12] or senescence [13]; (iv) the activation of autophagy, thereby supporting tumor cell survival [14]; (v) the induction of angiogenesis [15], and (vi) the promotion of invasion and metastasis [16]. On the other hand, hypoxia is also linked to an enhanced resistance towards anticancer treatments in clinical use, such as radiotherapy (RT) or chemotherapy (CT) [4,17], as further discussed below.

Notably, although the pronounced effects of hypoxia on cancer cell biology are well established, they are not taken into account in standard cell culture experiments, where cells are usually incubated at approximately 20% O_2_ (corresponding to 95% air composed of 79% N_2_ and 21% O_2_, plus 5% CO_2_). Although called “normoxia”, these conditions do not reflect the median O_2_ content of normal tissues (“physoxia”), which lies around 5–6% O_2_ in most organs, with some exceptions [1,5]. Importantly, due to the diffusion and perfusion limitations discussed above, tumors often are substantially less oxygenated than the corresponding normal tissue, and many cancer entities exhibit median O_2_ concentrations of less than 2% [1,5]. These considerations raise the question whether our conceptions of carcinogenesis that are derived from standard cell culture conditions are reflected under the low oxygen concentrations as they are present in the hypoxic sub-regions of many cancers.

## 3. Human Papillomaviruses and Cancer

At least 20% of the total cancer incidence in humans is attributable to infections [18]. In this context, oncogenic HPV types play a prominent role, since approximately every fourth of the infection-linked cancers worldwide is caused by this group of viruses [19]. HPV-induced cancers include cervical carcinomas, which alone account for more than 500,000 new cancer cases and over 250,000 cancer deaths per year [20]. Nearly 100% of cervical cancers are HPV-positive, with HPV16 (ca. 55–60%) and HPV18 (ca. 10–15%) being the most frequent types [21]. A substantial number of additional anogenital malignancies are HPV-positive as well, such as carcinomas of the anus (88%), vagina (78%), vulva (25%), and penis (50%) [19]. Moreover, a significant fraction of HNSCCs are linked to HPV infections, in particular oropharyngeal cancers (OPCs), which, in highly industrialized countries (e.g., Western Europe and the U.S.A.), are HPV-positive in 70–80% of cases. Differing from the virus type distribution in cervical cancers, HPV-positive OPCs contain HPV16 sequences in approximately 95% of cases [19,22].

In the process of HPV-induced carcinogenesis, the viral E6 and E7 oncoproteins target crucial tumor suppressor pathways for functional inactivation. For example, E6 forms a trimeric complex with the cellular ubiquitin ligase E6AP (E6-associated protein) and the p53 tumor suppressor protein [23], eventually leading to the proteolytic degradation of p53 [24]. The E7 oncoprotein binds to and inactivates the retinoblastoma tumor suppressor protein pRb [25]. Notably, the p53 and pRb pathways are also commonly impaired in HPV-negative human cancers by other routes, including somatic mutations of the *TP53* and *RB1* genes [26,27]. Thus, targeting the p53 and pRb proteins by the HPV oncoproteins represents an alternative strategy to block these key tumor suppressor pathways. Importantly, however, and in contrast to—for example—somatic mutations of the *TP53* and *RB1* genes, the inactivation of the p53 and pRb pathways by the HPV oncoproteins is reversible. In particular, the inhibition of E6/E7 expression in HPV-positive cancer cells leads to the reconstitution of p53 and pRb signaling and to the induction of cellular senescence [28,29,30,31,32], which is classically defined as an essentially irreversible growth arrest [33].

Consequently, it is widely assumed that HPV-positive cancer cells are “oncogene addicted” [34] in that they must continuously express E6/E7 in order to avoid the re-induction of dormant tumor suppressor pathways, such as p53 and pRb signaling. Notably, however, cervical cancers are often characterized by a heterogeneous distribution of hypoxic and better oxygenated sub-areas, and exhibit a median O_2_ concentration of only 1.2%. HNSCCs appear to be only slightly better ventilated, with median O_2_ concentrations between 1.3% and 1.9% [1,5,35]. The evidence that hypoxia can profoundly alter tumor cell biology raises the question whether the hypoxic conditions present in sub-regions of HPV-positive cancers may affect the crosstalk between the viral oncogenes and their host cell.

## 4. Regulation of Senescence in Normoxic and Hypoxic HPV-Positive Cancer Cells

A recent study investigated the effects of chronic hypoxia (1% O_2_) on HPV-positive cancer cells [36]. It was observed that viral E6/E7 expression is strongly repressed. Furthermore, in contrast to normoxia where E6/E7 repression results in a strong p53 and p21 upregulation, the downregulation of E6/E7 under hypoxia is not linked to an increase of p53 or p21 levels. Moreover, unlike under normoxic conditions, E6/E7 repression under hypoxia does not result in the induction of senescence in HPV-positive cancer cells. Instead, the cells react with a reversible proliferation stop that can be overcome by reoxygenation. These results indicate that hypoxic HPV-positive cancer cells have the potential to evade the presumed selection pressure to sustain viral oncogene expression, without undergoing senescence.

What is the mechanism underlying the discrepant phenotypic responses following E6/E7 repression in normoxic and hypoxic HPV-positive cancer cells? In normoxic HPV-positive cancer cells, the induction of senescence upon *E6*/*E7* silencing is linked to the reconstitution of the anti-proliferative p53 (and subsequently the p53-mediated stimulation of the negative cell cycle regulator p21) and pRb pathways [30,37]. This is in line with the well-documented pro-senescent potential of p53 and pRb signaling [33]. More recently, it has been found that the activity of the mechanistic target of rapamycin (mTOR) signaling cascade plays a critical role for the induction of senescence in many tumor cell models [38,39]. This is also the case for normoxic HPV-positive cancer cells, since treatment with chemical inhibitors of mTOR signaling, such as rapamycin or KU-0063794, allows the cells to evade senescence under conditions of efficient *E6*/*E7* silencing by RNA interference (RNAi) [36]. Collectively, these observations could be accommodated into a model [39] according to which the induction of senescence basically requires two major events: (i) a proliferative arrest (e.g., through the activation of the p53/p21 and/or pRb pathways) in the presence of conflicting growth promoting stimuli (e.g., through active mTOR signaling); and (ii) the conversion (“geroconversion”) of the reversible proliferation arrest to senescence, a process which can be driven by mTOR signaling.

The anti-senescent effects of the mTOR inhibitors in normoxic HPV-positive cancer cells also indicate that the mTOR pathway remains active in these cells despite strong E6/E7 downregulation. In line with this, and indicative for sustained mTOR signaling, the amounts of phosphorylated mTOR downstream targets are not (phospho-S6, phospho-p70-S6 kinase) or are only partially (phospho-4E-binding protein 1) reduced when *E6/E7* is silenced in HPV-positive cancer cells. This observation is also remarkable in the light of previous studies, which show that the HPV E6 protein stimulates mTOR signaling upon ectopic expression [40,41], including in primary human keratinocytes, the natural target cells for HPV infections [41]. It thus will be interesting to determine how mTOR signaling is maintained in normoxic cervical cancer cell lines when the endogenous E6/E7 expression is silenced.

Importantly, the mTOR pathway is strongly repressed under hypoxia in many cell systems [42], including cervical cancer cells [36]. This impairment of mTOR signaling is associated with the hypoxia-induced stimulation of REDD1 (regulated in development and DNA damage response 1) expression, which activates the TSC2 (tuberous sclerosis complex 2) protein, a negative regulator of mTOR signaling [43]. Blocking *REDD1* or *TSC2* expression by RNAi leads to the stimulation of mTOR signaling and to the emergence of senescent HPV-positive cancer cells under hypoxia [36]. Collectively, these data argue that the hypoxic impairment of mTOR signaling, which occurs at least in part via the inhibitory REDD1/TSC2 axis, enables HPV-positive cancer cells to evade senescence under conditions of E6/E7 repression.

## 5. HPV-Positive Cancer Cells under Hypoxia: Clinical Implications

Besides surgical excision, RT and radiochemotherapy (RCT) play central roles for the clinical management of HPV-positive cancers, such as cervical carcinomas and HNSCCs. CT alone usually has only limited therapeutic efficacy, and often is applied as a palliative treatment when surgery or RT are not possible, e.g., in patients with recurrent or metastatic disease [44,45,46]. Notably, both the general effects of tumor hypoxia as well as the specific hypoxic response of HPV-positive cancer cells bear the potential to increase resistance towards all of these treatment strategies.

In particular, hypoxia is considered to be a major obstacle for the therapeutic efficacy of RT, mainly due to the fact that O_2_ is required to manifest the DNA lesions that are induced by ionizing radiation [47,48]. As a consequence, hypoxic cells exhibit an approximately three-fold increase in their resistance towards RT compared with well-oxygenated cells [47,48]. The presence of hypoxic sub-regions in tumors can also cause resistance against CT, for example (i) as a consequence of the limited perfusion-dependent delivery of chemotherapeutic drugs in these areas; (ii) through the induction of genes that can protect tumor cells against CT, such as the *MDR1* (multidrug resistance 1) gene [49]; and (iii) through a hypoxia-linked inhibition of cancer cell proliferation, since many chemotherapeutic agents are preferentially active against dividing cells [50]. Thus, the proliferative stop of HPV-positive cancer cells that is observed under hypoxia could contribute to their resistance towards CT. It further should be noted that CT exerts its anti-tumorigenic properties not only by eliminating cancer cells via apoptosis, which could be attenuated by the anti-apoptotic effects of hypoxia [12], but also by inducing cellular senescence [51,52,53]. However, this latter activity could be counteracted by the hypoxia-induced impairment of mTOR signaling, which not only protects hypoxic HPV-positive cancer cells from the pro-senescent effects of E6/E7 inhibition, but also from pro-senescent CT [36].

Besides these classical treatment regimens, there are intense efforts to develop novel therapeutic strategies targeting E6/E7 in HPV-positive cancers. The theoretical basis for most of these approaches is the conception that the HPV oncoproteins are regularly expressed in HPV-positive cancer cells and that they play a crucial role in the maintenance of their malignant phenotype. Accordingly, antigens derived from the viral oncoproteins are believed to represent attractive targets for immunotherapy of cervical cancer or HPV-positive HNSCCs, since HPV-positive cancer cells would be unable to downregulate E6/E7 expression as an immune evasion mechanism [54,55]. However, this could be an oversimplified view, since hypoxic HPV-positive cancer cells can efficiently shut down E6/E7 expression [36], and consequently repress viral antigen production. Moreover, the hypoxic microenvironment itself is known to exert immunosuppressive effects by affecting the activities of various types of immune cells. These include myeloid-derived suppressor cells (MDSCs), regulatory T cells (Tregs), and tumor-associated macrophages (TAMs) that have been linked to the suppression of an effective anti-tumor immune response under hypoxia [56,57,58]. Thus, both viral oncogene repression and general immunosuppression in hypoxic tumor sub-regions could be major obstacles for the efficacy of immunotherapeutic approaches targeting E6- or E7-derived antigens, which, in most instances, have thus far been rather limited with regard to their therapeutic benefit [54,59].

Furthermore, the rapid and efficient senescence response that is observed upon E6/E7 repression in normoxic HPV-positive cancer cells raises the possibility that inhibitors of E6/E7 expression or function possess therapeutic potential for the treatment of HPV-positive tumors [28,29,30,31,32,60]. Indeed, there is evidence that the induction of senescence in tumor cells (“pro-senescence therapy”) holds promise as a therapeutic anticancer strategy [61,62,63]. However, there are also potential pitfalls associated with this approach. Firstly, some observations suggest that the senescent phenotype may not be completely stable under all conditions. For example, the cellular senescence and concomitant growth arrest induced by RT or CT may not be as irreversible as it is in its classical definition, and rare tumor cells evading from this regulation could grow even more aggressively and exhibit an increased therapeutic resistance [53,64,65]. Secondly, whereas the induction of stable senescence in cancer cells would be therapeutically desirable, cells in the tumor microenvironment (such as stromal fibroblasts) that also senesce in response to CT or RT can acquire a “senescence-associated secretory phenotype (SASP)” [33], leading to the secretion of both anti-tumorigenic and pro-tumorigenic factors [33,66]. Thus, as a result of the pro-tumorigenic potential of the SASP, senescent cells in the tumor microenvironment may actually augment the oncogenicity of cancer cells that have escaped from the pro-senescent or pro-apoptotic effects of CT or RT, by supporting their proliferation and survival and by increasing their metastatic potential [66]. In line with this possibility, mTOR inhibitors that can interfere with the secretion of major components of the SASP have been reported to sensitize tumors towards CT [67,68].

In view of the potentially pro-tumorigenic effects of the SASP of surrounding stromal cells, it thus might be beneficial to induce senescence selectively in tumor cells. In principle, this specificity could be achieved by interfering with E6/E7 expression or function, since these therapeutic targets are not present in normal (HPV-negative) cells. Unfortunately, however, hypoxic HPV-positive cancer cells would be expected to resist a pro-senescence therapy that is based on E6/E7 inhibition, since the expression of the therapeutic targets is blocked and pro-senescent mTOR signaling is impaired [36].

Yet, it should be emphasized that these considerations do not preclude a therapeutic use of prospective E6/E7 inhibitors, since they would be expected to act in a pro-senescent way in non-hypoxic HPV-positive cancer cells where the therapeutic targets are expressed and mTOR signaling is active. The inhibition of E6/E7 could thus be combined with treatment strategies currently under development that aim to attack hypoxic cancer cells [4]. These include agents which interfere with the unfolded protein response (UPR), a mechanism that contributes to tumor cell survival under hypoxia, or substances which block HIF-1α and HIF-2α (hypoxia-induced factors 1α and 2α)-linked signaling pathways that adapt tumor cells to hypoxia, e.g., by inducing metabolic reprogramming, enhancing tumor cell survival, and supporting angiogenesis and metastasis [4,69]. In addition, although their therapeutic efficacy in the clinic has been mostly disappointing thus far, much hope is also put on the development of improved hypoxia-activated prodrugs (HAPs) that are metabolized to cytotoxic agents under hypoxic conditions [70]. Other approaches to target the hypoxic sub-regions of cancers include the application of certain bacteria, such as the oxygen gradient-sensing *Magnetococcus marinus* strain MC-1 that can transport drug-containing nanoliposomes into hypoxic tumor regions [71], or *Clostridium novyi*-NT that spreads to and destructs hypoxic cancer areas upon intratumoral injection [72].

Notably, the phenotype of hypoxic HPV-positive tumor cells may not only provide therapeutic resistance, but could also be a risk factor for tumor recurrence. In particular, by their ability to reinduce cellular proliferation upon reoxygenation, hypoxic HPV-positive cancer cells may serve as a reservoir for tumor regrowth when their oxygen supply is increased. This can occur, for example, through neoangiogenesis [73], or following the therapeutic shrinkage of tumors [74].

## 6. Conclusions and Perspectives

**Figure 2:**
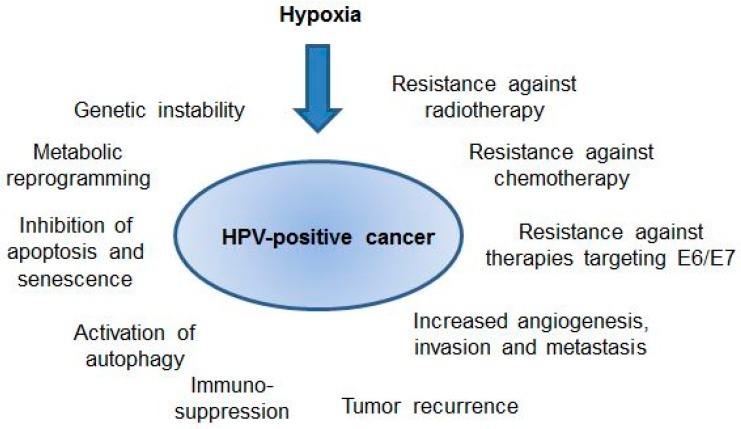
Potential effects of hypoxia on the biology and clinical behavior of human papillomavirus (HPV)-positive cancers. For further details please refer to the text.

Hypoxia has the potential to strongly affect the biology of HPV-positive cancers, both through its general effects in tumors as well as through the modulation of the interplay between oncogenic HPVs and their host cell (Figure 2). In the latter context, hypoxic HPV-positive cancer cells can induce a state of dormancy, which is characterized by a shutdown of viral oncogene expression, an impairment of mTOR signaling, and a reversible growth arrest. Hypoxic HPV-positive cancer cells thus can escape from key regulatory principles that have been observed under normoxia, with consequences for both their cellular phenotype and therapeutic susceptibility. However, important questions remain to be solved.

Firstly, which molecular mechanisms are responsible for the strong inhibition of *E6*/*E7* oncogene expression under hypoxia? Secondly, why are the p53 levels not restored in hypoxic HPV-positive cancer cells, although E6 is repressed? Thirdly, how is mTOR signaling maintained in normoxic HPV-positive cancer cells under conditions of *E6*/*E7* silencing and cellular growth inhibition? Fourthly, since the impairment of mTOR signaling is crucial for the ability of hypoxic HPV-positive cancer cells to evade senescence, will it be possible to identify agents which override this regulatory principle and thereby induce senescence in an mTOR-independent manner? Could they be of therapeutic value in combination with RT, CT, RCT, or E6/E7 inhibition, which preferentially act on non-hypoxic cells? Fifthly, in view of the fact that standard cell culture conditions (20% O_2_) do not reflect the O_2_ levels in normal cervical tissue (median concentration of 5.5% O_2_) [1,5], are there differences in the phenotypic responses listed above between HPV-positive cancer cells that are grown under physoxia instead of normoxia?

Moreover, it should be noted that analyses of the crosstalk between the viral *E6/E7* oncogenes and their host cell thus far have focused on the effects of chronic hypoxia (1% O_2_ over days) [36]. However, there is evidence that cycling hypoxia (Figure 1B) may also be highly relevant in terms of increased tumor aggressiveness and enhanced therapeutic resistance [6,7,75]. Cycling hypoxia leads to a huge rise of ROS production, and paradoxically to a strong activation of HIF-1 signaling during the reoxygenation phases [76]. The latter observation is explained by experimental evidence that HIF-1-regulated transcripts are kept in cellular stress granules under hypoxia, which disaggregate upon reoxygenation and allow for the translation of the HIF-1-regulated RNAs [76]. Enhanced HIF-1 signaling and/or increased ROS production affect a broad range of different cancer-linked processes, such as the control of cell proliferation, apoptosis, senescence, cellular metabolism, genetic stability, angiogenesis, and metastasis. This can culminate in more aggressive cancer growth and higher therapeutic resistance (reviewed in [69,77]). Interestingly, in a transgenic mouse model of HPV-induced cervical carcinogenesis, enhanced HIF-1α expression resulted in an increase of tumor cell proliferation and invasion [78]. This finding indicates a possible cooperation between the HPV oncogenes and HIF-1α during cervical cancer progression. It thus should be informative to investigate the effects of cycling hypoxia on the expression of HIF-1α and the HPV oncogenes in HPV-positive cancer cells, and the resulting consequences for their cellular phenotype and therapeutic sensitivity. The further elucidation of the crosstalk between oncogenic HPVs and their host cell under hypoxia may therefore not only increase our current understanding of the molecular mechanisms of HPV-induced carcinogenesis, but also be revealing in regards to the clinical behavior of HPV-positive cancers and their therapeutic resistance.
